# Delineating the roles of HOXB13 X285K variant in prostate cancer

**DOI:** 10.17161/sjm.v2i1.23160

**Published:** 2025-01-14

**Authors:** Xiaodong Lu, Samantha Ye, Mingyang Liu, Jindan Yu

**Affiliations:** 1 Department of Urology, Emory University School of Medicine, Atlanta, GA, USA;; 2 Department of Human Genetics, Emory University School of Medicine, Atlanta, GA, USA;; 3 Winship Cancer Institute, Emory University School of Medicine, Atlanta, GA, USA;

**Keywords:** prostate cancer, HOXB13, X285K

## Abstract

*HOXB13* X285K is a frameshift mutant caused by the deletion of one nucleotide, c.853delT, within the *HOXB13* stop codon, resulting in an extension of HOXB13 protein by 96 amino acids on the C terminus. It was found primarily in men of West African ancestry and associated with early-onset prostate cancer (PCa) and more advanced stage. Whether and how X285K contributes to PCa progression remains largely unknown. Here, we established isogenic 22Rv1 cell lines with heterozygous wildtype/X285K HOXB13, which recapitulates the genotypes of X285K in PCa patients. In addition, using the unique C-terminal region of X285K as an antigen, we developed an antibody that specifically recognizes the HOXB13 X285K protein. Lastly, we demonstrated that X285K, similar to wildtype HOXB13, was able to rescue the effects of *HOXB13* knockdown on both induced and repressed genes, such as *FASN* and *PSA*, in PCa cells. In summary, our study reports that X285K retained the transcriptional regulation ability of wildtype HOXB13 and provides isogenic PCa cell lines with wildtype and/or X285K HOXB13 expression and an X285K-specific antibody for a comprehensive investigation of X285K function in PCa.

## Introduction

HOXB13, a member of homeobox-containing transcription factors, is predominantly expressed in the prostate, and to a much lesser degree, in the colon [1, 2]. Disruption of the homeobox domain of HOXB13 impairs ventral prostate lobe development in transgenic mouse models [3]. HOXB13 is highly expressed in human benign prostatic hyperplasia (BPH) and primary prostate cancer (PCa); however, ~30% metastatic castration-resistant prostate cancer (CRPC) and most AR-negative PCa express low levels of HOXB13 [4–6]. Studies have shown that dysregulation of HOXB13 promotes PCa progression through both androgen receptor (AR) dependent- and -independent mechanisms [4, 5, 7, 8]. For example, HOXB13 is a key determinant of androgen response in androgen-dependent LNCaP PCa cell line, wherein HOXB13 modulates AR activity through facilitating, recruiting, or repressing AR binding to cis-regulatory elements in the genome [7]. In addition, HOXB13 reprograms AR cistrome from normal- to tumor-specific binding sites when co-expressed with pioneer transcription factor FOXA1 in prostate cells[8]. In CRPC models, such as 22Rv1 and LN95, HOXB13 mainly co-localizes with AR-V7, a CRPC-associated AR variant, rather than full-length AR (AR-FL), to govern AR-V7-driven oncogenic programs, including cell proliferation[5]. On the other hand, we have previously reported that HOXB13 suppresses lipogenic programs in PCa via HDAC3/NCoR-mediated epigenetic reprogramming, independently of AR, in both androgen-sensitive and CRPC cells [4]. Loss of HOXB13 promotes prostate tumor metastasis by activating the *de novo* lipid synthesis process [4].

Somatic mutations of *HOXB13* in PCa are rare. However, multiple germline variants have been identified in the *HOXB13* gene in men of different ancestral populations, such as G84E in European ancestry [9], G135E in Chinese ancestry[10], and X285K in African ancestry [11–14]. The prevalence of G84E mutation in European descent who had PCa is 1.4%, significantly higher than the 0.1% in control subjects [9]. PCa patients carrying G84E mutation exhibit high-risk disease, including relatively early onset, higher prostate-specific antigen (PSA) levels, and Gleason scores at diagnosis [15]. Importantly, a recent study reported that fatty acid synthase (FASN) expression is significantly elevated in PCa from carriers of G84E mutation compared with matched controls [16]. These are consistent with our earlier report that *HOXB13* G84E mutation induces the expression of PSA and lipogenic enzymes, such as FASN, by disrupting the interaction between HOXB13 and transcriptional repressor complex HDAC3/NCoR, and leads to tumor metastasis [4, 16].

*HOXB13* X285K is a frameshift variant due to the deletion of one nucleotide, c.853delT, within the *HOXB13* stop codon, resulting in an extension of HOXB13 protein by 96 amino acids (aa), and has been associated with younger age at PCa diagnosis and high-grade Gleason Score of PCa [11]. A recent study has started to delineate the functional consequences of this mutation and found that X285K is a gain-of-function mutation resulting in increased E2F/MYC signature through enhanced binding at target promoters/enhancers of these genes [12]. However, whether the mutation alters the previously reported functions of HOXB13 remains to be addressed. To this end, here we established isogeneic 22Rv1 cell lines expressing *HOXB13* wild-type (WT), WT/X285K heterozygote (Het), and X285K homozygote (Homo) using clustered regularly interspaced short palindromic repeats (CRISPR) technology. Further, we generated an antibody (anti-X285K) that specifically recognizes the C-terminal 96 aa that is unique to HOXB13 X285K and validated the specificity of this anti-X285K antibody for western blot (WB), immunoprecipitation (IP) and immunofluorescence (IF) applications. Lastly, we demonstrated that X285K retains the function of HOXB13 in repressing known target genes such as FASN and PSA.

## Results

### Generation of isogenic PCa cell lines with HOXB13 X285K mutation

**Figure 1.**
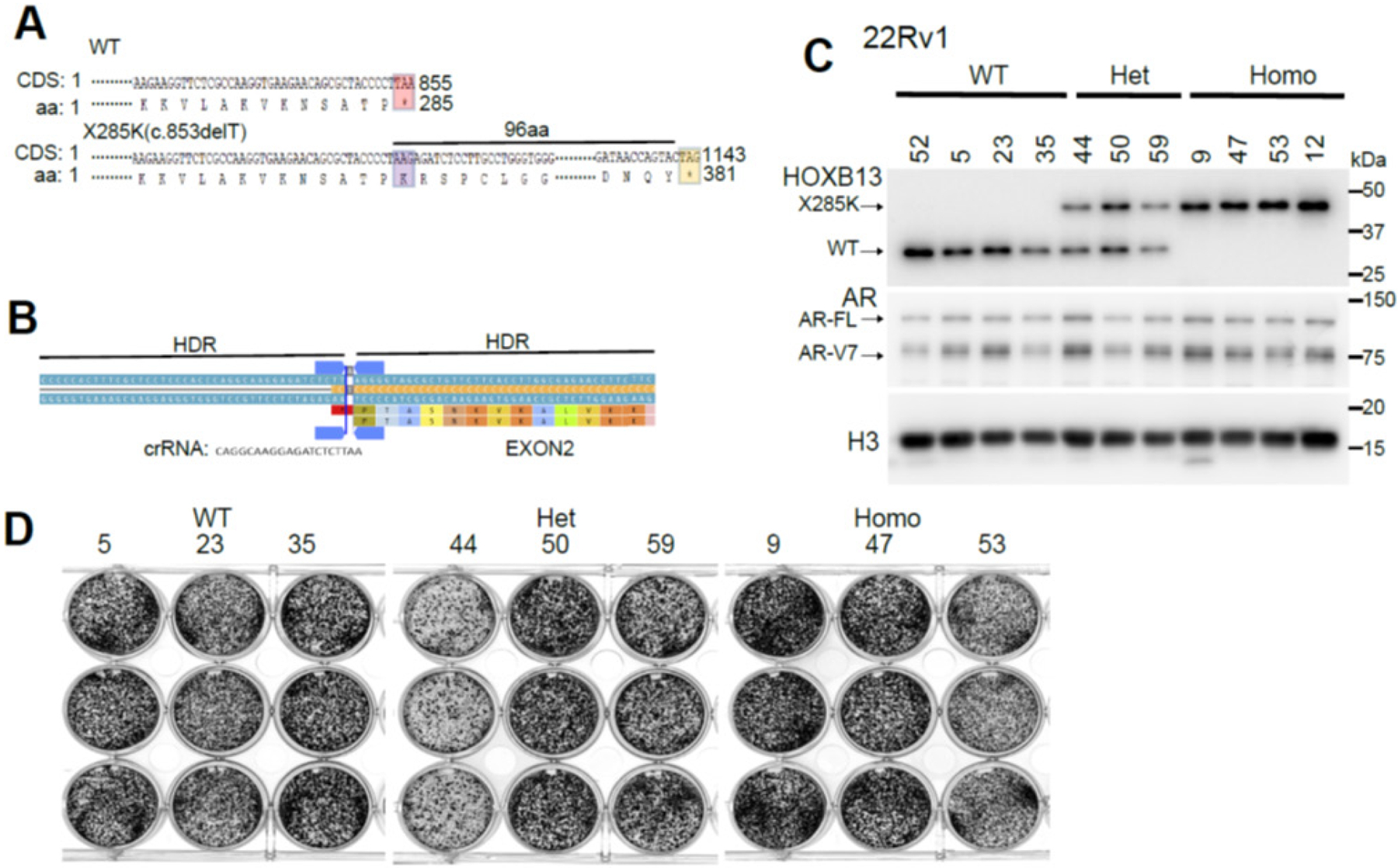
Generation of 22Rv1 isogenic cell lines with *HOXB13* X285K mutation using CRISPR **A**. Schematic of HOXB13 wild-type (WT) and nonstop extension mutation X285K. The stop codons in WT and X285K were highlighted in pink and yellow, respectively; aa.285K (TAA into AAG) in X285K was highlighted in purple. CDS: coding sequence; aa: amino acids, **B**. Schematic of crRNA and homology-directed repair (HDR) template designs for c.853delT in *HOXB13* stop codon. **C**. Western blot (WB) analysis of HOXB13 expression in 22Rv1 isogenic clones with WT, WT/X285K heterozygote (Het), and X285K/X285K homozygous (Homo) *HOXB13*. An antibody targeting the N-terminal of HOXB13 was used to detect both HOXB13 WT and X285K **D**. Colony formation assays of 22Rv1 isogenic clones.

*HOXB13* X285K is a heterozygous germline variant detected specifically in clinical PCa patients of African ancestry [13]. Analyses of reference sequence revealed that X285K mutation would lead to a single nucleotide (T) deletion in the stop codon of the wildtype (WT) HOXB13 protein, resulting in continued protein translation until it reaches the next in-frame stop codon, which is 96 amino acids (aa) down the road (Fig.1A). Whether the *HOXB13* allele containing c.853delT mutation (X285K) is translated to express the desired protein in PCa remains unknown. To address this and model the genetic status of HOXB13 X285K mutation in human PCa, we utilized CRISPR technology to edit the HOXB13 gene locus to generate isogenic cell lines of wildtype (WT), WT/X285K heterozygous (Het), and X285K/X285K homozygous (Homo) *HOXB13* (Fig.1B). We used 22Rv1 for CRISPR editing because there are two copies of chromosome 17 in 22Rv1 cells, compared to LNCaP and C4–2B that are polyploidy, which make it more difficult to delete the target region from all copies of chromosomes completely. Single clones were selected and grew out after CRISPR editing. Critically, WB analysis using an antibody recognizing the N-terminal of HOXB13 (N-terminal) detected the expected single band of ~33kDa of WT HOXB13 in the WT clones (Fig.1C). Critically, a single band corresponding to a protein of ~11kDa larger than WT HOXB13 was detected in the Homo clones, suggesting that X285K mutation indeed led to a mutant protein with expected 96aa extension. Accordingly, two bands of ~33 and ~44 kDa, corresponding to WT and X285K HOXB13, respectively, were detected in the 3 WT/X285K (Het) clones (Fig.1C). Of note, the expression levels of HOXB13 WT and/or X285K varied considerably across the various clones, and so did AR and AR-V7 levels, reflecting clonal variations. Consistent with this, the growth rates of individual clones were variable, and there were no significant differences between WT and mutant groups of clones (Fig.1D). In summary, we have successfully generated isogenic 22Rv1 clones of WT, WT/X285K, or X285K homo alleles and demonstrated that the X285K mutation led to the expression of a HOXB13 mutant protein of a larger molecular weight, being consistent with the predicted stop codon extension.

### Development of an HOXB13 X285K (anti-X285K)-specific antibody

**Figure 2.**
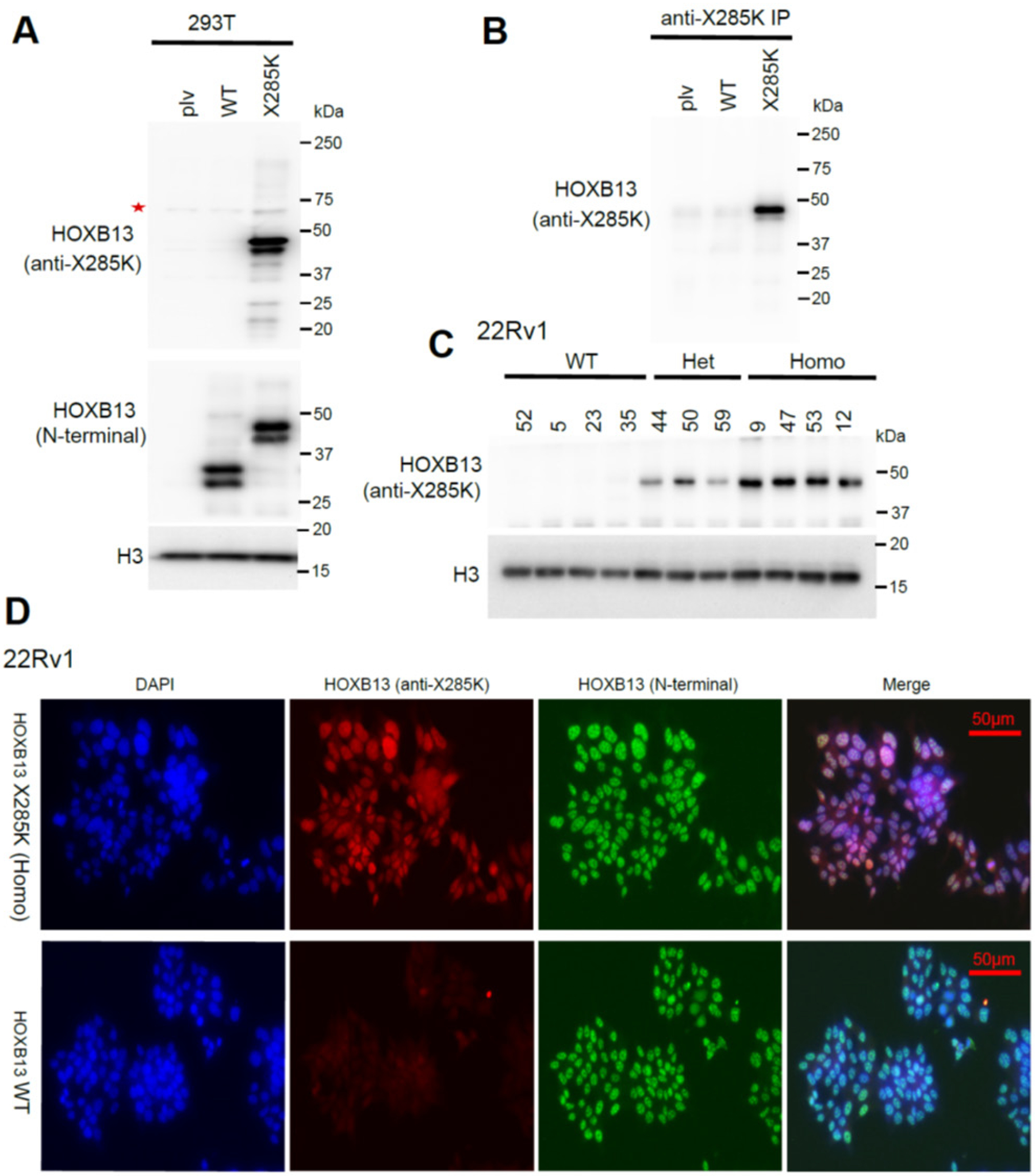
Characterization of an anti-HOXB13 X285K (anti-X285K)-specific antibody **A**. WB analysis of the specificity of anti-HOXB13 X285K antibody. 293T cells were transfected with control (plv), HOXB13 WT or X285K, and subjected to WB at 48h post-transfection. An N-terminal-targeting antibody was used to detect both HOXB13 WT and X285K. The red star indicates non-specific band. **B**. Immunoprecipitation (IP) analysis demonstrated the anti-X285K antibody works for IP applications. The lysate from 293T cells transfected with control (plv), HOXB13 WT or X285K were subjected to IP using anti-X285K antibody. **C**. WB analysis of the specificity of anti-X285K antibody in 22Rv1 isogenic clones as shown in Fig.1C. **D**. Immunofluorescence (IF) analysis demonstrated the anti-X285K antibody works for IF applications. The IF experiments were performed in isogenic 22Rv1 cells with WT (clone5) or X285K/X285K (Homo, clone 47) HOXB13. An N-terminal-targeting antibody was used to detect both HOXB13 WT and X285K.

To further validate that the larger HOXB13 protein detected in the X285K-mutant cells is indeed the X285K mutant, we generated an antibody (anti-X285K) using the C-terminal extended 96aa as antigen. WB analysis of 293T cells with ectopic expression of control, HOXB13 WT or X285K demonstrated that the antibody specifically recognizes X285K, but not WT HOXB13 (Fig.2A). By contrast, an N-terminal-targeting antibody detected both WT and X285K proteins, as expected. In addition, we demonstrated that our newly generated X285K-specific antibody could be useful for immunoprecipitation (IP) to pull down X285K protein, which was expressed in 293T cells through transient transfection (Fig.2B). Next, to evaluate whether the anti-X285K antibody can recognize the X285K protein expressed in the isogenic 22Rv1 cell lines that recapitulate the genetic status of *HOXB13* X285K in human PCa[13], we performed WB analysis in 22Rv1 isogenic clones mentioned earlier. WB data clearly showed that the anti-X285K antibody detected X285K proteins in Het and Homo, but not WT, clones (Fig.2C). Lastly, we tested whether the antibody works for IF assay. Importantly, IF staining showed that the anti-X285K antibody detected a strong signal in the nuclei in Homo but not WT clones and co-located with signals detected by an antibody targeting the N-terminal of HOXB13, which detected both X285K and WT HOXB13 proteins, as expected (Fig.2D). Therefore, we have successfully generated an antibody that specifically recognizes the X285K-HOXB13 protein and showed that the antibody could be useful for WB, IP, and IF analyses of X285K HOXB13 specifically.

### HOXB13 X285K mutant retained the ability to suppress target genes of WT HOXB13

**Figure 3.**
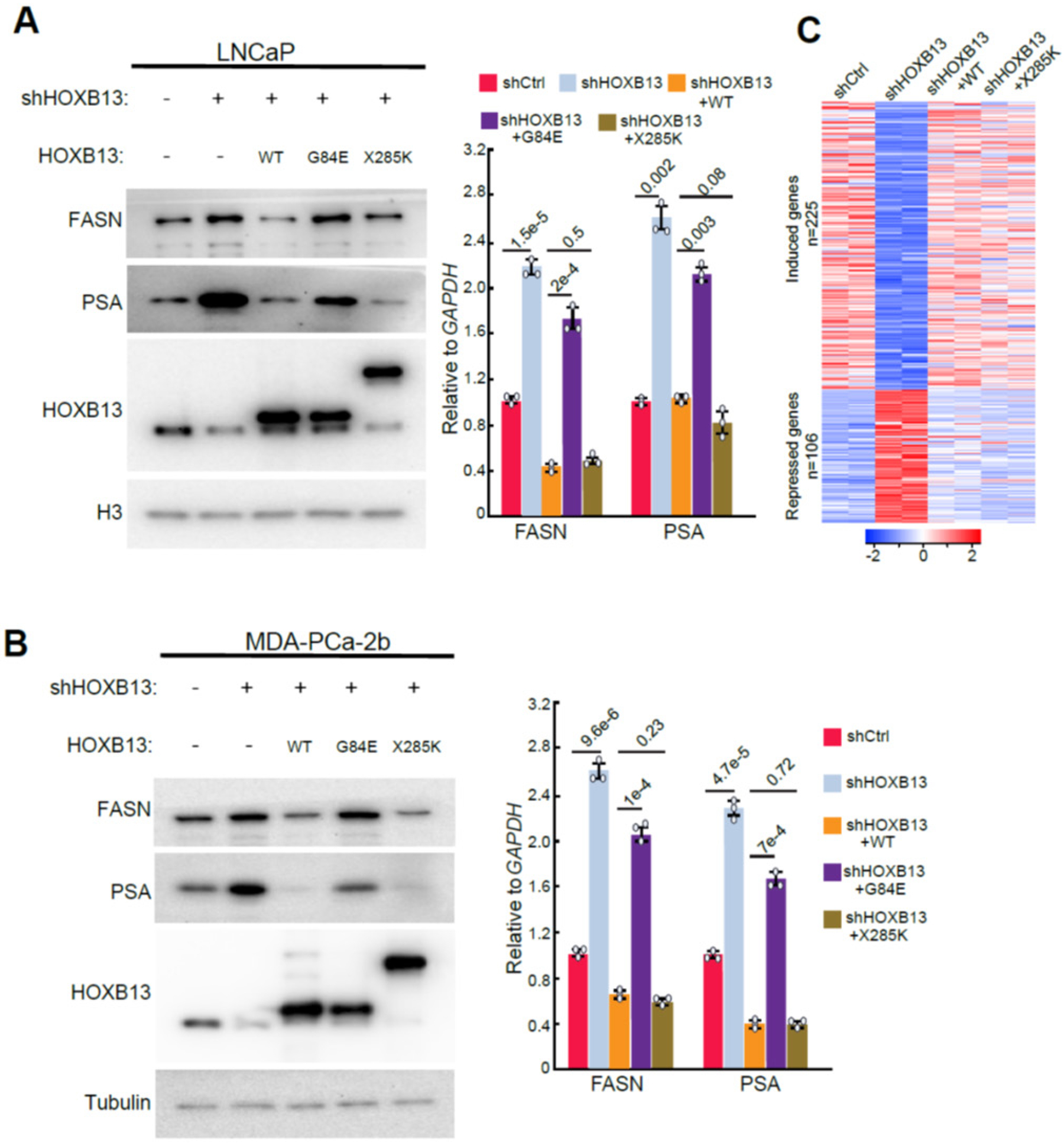
HOXB13 X285K mutant retained the ability to repress target genes of WT HOXB13. **A-B**. WB (left) and RT-PCR (right) analysis of FASN and PSA regulation by HOXB13 X285K in LNCaP (**A**) and MDA-PCa-2b (**B**). RT-PCR data were normalized to GAPDH and shown as technical replicates from one of three (n = 2) bio-logical replicates. Data shown are mean ± s.e.m. *P* values by unpaired two-sided *t*-test. **C**. Heatmap showing HOXB13 target gene regulation by HOXB13 X285K in LNCaP cells. RNA-seq was performed in duplicate of the indicated cells. HOXB13 target genes were identified by DESeq2 with fold change (FC)≥2, adjusted *p*<0.05. Color bar: z-score.

Our previous study has shown that PSA and FASN are HOXB13-target genes in that HOXB13 directly binds to their promoters to repress their expression[4]. This important function was disabled by G84E mutation, which is located at the MEIS domain where HOXB13 interacts with the HDAC3/NCoR corepressor complex. We wondered whether the extension of the HOXB13 protein in the X285K mutant affects its ability to repress target genes. To this end, we performed HOXB13 WT, G84E, and X285K rescue in LNCaP cells with *HOXB13* knockdown (KD). Consistent with our previous report [4], knockdown of HOXB13 significantly induced FASN and PSA expression, which were repressed by the re-expression of ectopic WT, but not G84E, HOXB13. Interestingly, similar to WT HOXB13, X285K also markedly repressed FASN and PSA repression (Fig.3A), suggesting maintenance of this important function of HOXB13. As X285K mutation was only identified in PCa patients of African ancestry, we repeated the experiment in MDA-PCa-2b, a PCa cell line derived from an African PCa patient. Critically, WB analyses confirmed that X285K could repress FASN and PSA expression, similar to WT, but distinct from G84E mutant (Fig.3B). To evaluate the global effects of X285K in regulating *HOXB13*-target genes, we performed duplicate RNA-seq analyses of LNCaP cells with *HOXB13* KD and/or rescue by WT or X285K *HOXB13*. We identified 225 and 106 genes that were respectively down- and up-regulated upon *HOXB13* KD, with fold change (FC)≥2 and adjusted p<0.05 (Fig.3C). Not surprisingly, these effects were fully rescued by the re-expression of WT HOXB13 in the HOXB13-KD cells. Critically, our data revealed that X285K could also rescue most of these effects, suggesting that X285K remained proficient in transcriptional regulation of target genes, largely similar to WT HOXB13.

## Discussion

*HOXB13* X285K variant was reported in 1.01% of PCa patients of self-reported Black ancestry, vs. 0.01% in those of White ancestry [12]. The variant mutation is likely to have significant functional consequences, as it extends the HOXB13 transcription factor protein by 96aa on the C terminus, and clinically, it has been associated with early-onset PCa and high-grade PCa [11]. However, very few studies have addressed its function and the underlying mechanisms, largely due to the lack of appropriate models and reagents. Here, we established multiple *HOXB13* WT/X285K heterozygous 22Rv1 clones to recapitulate the genetic background of *HOXB13* X285K in PCa patients. In addition, we successfully generated an anti-HOXB13 X285K-specific antibody that works for WB, IP, and IF applications. These will be great resources for the research community to further understand X285K mutation in PCa progression. For instance, the X285K-specific antibody could be utilized to detect and validate X285K expression in clinical PCa samples, which is important as a previous study has utilized allele-specific transcription to show that five out of seven G84E carriers transcribed only the wild-type allele, despite the presence of G84E allele [17]. Critically, in our isogenic 22Rv1 cells, we detected both WT and X285K expression in the heterozygous clones, supporting that X285K protein was indeed expressed from the mutant allele. This, however, needs to be validated in PCa patient samples.

We did not observe a clear loss/gain of function of X285K in the regulation of prototype HOXB13-target genes *PSA* and *FASN* and global transcriptional targets, in contrast to a clearly impaired function of the G84E mutation. HOXB13 represses PSA and FASN by interacting with the HDAC3/NCoR co-repressor complex via its MEIS domain to recruit them to the target enhancers [4]. It is very likely that the extension of HOXB13 protein on the C terminus in X285K does not affect its interaction with HDAC3/NCoR. Genome-wide analyses, such as RNA-seq, ChIP-seq, and mass spectrometry analyses of various PCa models, are required to determine how X285K mutation alerts global gene expression, HOXB13 binding, and cofactor interactions. Interestingly, a recent study reported that the chromatin binding affinity of X285K was increased compared with WT in LN95 cells to induce the expression of E2F/MYC genes [12]. Whether there is a global enhancement of HOXB13 cistrome or a reprogramming awaits further clarification. The isogenic 22Rv1 cell lines we generated in this study will be a great resource to determine target cistromes, downstream genes, and interacting proteins of X285K through comparative RNA-seq, ChIP-seq, and mass spectrometry analyses of control and HOXB13-knockdown cells. Although our data thus far did not reveal a loss/gain of function of X285K in suppressing PSA and FASN, C-terminal extension of a protein has been previously shown to alter protein stability, its subcellular localization, post-translational modifications, and its interaction with cofactor proteins [18]. Our data showed that X285K remained to be localized in the nuclei of PCa cells, but this is yet to be confirmed in clinical samples. It would also be exceedingly interesting to determine other potential effects of the mutation on HOXB13 protein in future studies utilizing the reagents we generated in this study.

## Materials and Methods

### Cell lines and antibodies

PCa cell lines LNCaP, MDA-PCa-2b, and human embryonic kidney cell line HEK293T, cells were obtained from the American Type Culture Collection (ATCC) and cultured in either RPMI 1640 or DMEM with 10% FBS, 1% penicillin and streptomycin. Cell lines were either newly acquired from ATCC or authenticated within 6 months of growth and cells under culture are frequently tested for potential mycoplasma contamination. The information of antibodies used in this study: HOXB13 (Cell Signaling Technology, Cat#90944T), PSA (Cell Signaling Technology, Cat#2475T), FASN (Santa Cruz Biotechnology, sc-48357) c-MYC (Abcam, ab32072) for WB application; HOXB13 (Santa Cruz Biotechnology, sc-28333) for IF application.

### Plasmid constructs and lentivirus

The pGIPZ lentiviral shRNA targeting *HOXB13* (Clone ID: V3LHS_403019) and control vector were purchased from Open Biosystems. *HOXB13* WT, G84E and X285K were cloned into pLV-N-2HA (modified pLV-EF1a-IRES-Puro vector, Biosettia) by in-fusion cloning strategy (Takara, Cat#638943). All the plasmids were verified by sanger sequencing. For the generation of lentivirus, HEK293T cells were transfected with psPAX2 and pMD2G with target gene at ratio 2:1:1. Lentiviruses were collected at 48 h after transfection and filtered with a 0.45 μm filter. Lentiviruses, supplemented with 8 μg/ml polybrene, were used to infect PCa cells. Infected cells were collected for WB analysis on day5 of post-infection.

### Generation of isogenic 22Rv1 cell lines with HOXB13 X285K mutation

Alt-R^™^ HDR Donor Oligo and crRNA for c.853delT within *HOXB13* stop codon were designed using Alt-R HDR Design Tool from Integrated DNA Technologies (IDT). Transfection was performed in 22Rv1 cells by following the protocol from IDT: Alt-R CRISPR-Cas9 System: Cationic lipid delivery of CRISPR ribonucleoprotein complex into mammalian cells. 24h after transfection, the cells were dissociated into single cells using Accutase^®^ cell detachment solution (Innovative Cell Technologies) and sorted 1 cell/well into 96-well plates using fluorescence-activated cell sorting (FACS) or limiting dilution assay. The cells were harvested for WB analysis and genomic DNA extraction when the individual clones were ready. The clones were validated using an antibody targeting N-terminal of HOXB13.

### Generation of anti-HOXB13 X285K (anti-X285K) specific antibody

The customized antibody against HOXB13 X285K was generated from ABclonal. The extended 96 amino acids of HOXB13 X285K were used as antigens for antibody generation.

### RNA extraction, RT-PCR and RNA-seq analysis

RNA was extracted using the nucleospin RNA kit (Takara) according to the manufacturer’s recommended protocol. Then, 500 ng of RNA was reverse transcribed into complementary DNA (cDNA) using ReverTra Ace qPCR RT Master Mix kit (Diagnocine) following the manufacturer’s recommended protocol. qPCR was performed using 2X Universal SYBR Green Fast qPCR Mix (Abclonal, Cat#RK21203) and QuantStudio 3 (Thermo Fisher). For RNA-seq library preparation, total RNA was isolated as described above and performed in replicate or triplicate. 0.5 μg of high-quality-DNA-free RNA was used for library preparation (NEBNext Ultra RNA Library Prep Kit) according to the manufacturer’s instructions. The libraries passing quality control (equal size distribution between 250 and 400 bp, no adapter contamination peaks, no degradation peaks) were quantified using the Library Quantification Kit from Illumina (Kapa Biosystems, KK4603). Libraries were pooled to a final concentration of 10 nM and sequenced paired-end using the Illumina NovaSeq 6000. Human prostate cancer cell RNA-seq reads were mapped to NCBI human genome GRCh38. Raw counts of genes were calculated by STAR. FPKM values (fragments per kilobase of transcript per million mapped reads) were calculated by in-house Perl script. Differentially expressed genes (DEGs) were identified by DESeq2 with the default Wald test in a pairwise manner.
